# Validity and reliability of the Japanese version of the Substance Use Stigma Mechanism Scale

**DOI:** 10.1371/journal.pone.0310514

**Published:** 2024-10-31

**Authors:** Ayumi Takano, Chiaki Hiraiwa, Erina Oikawa, Akiko Tomikawa, Kyosuke Nozawa

**Affiliations:** 1 Department of Drug Dependence Research, National Institute of Mental Health, National Center of Neurology and Psychiatry, Tokyo, Japan; 2 Department of Mental Health and Psychiatric Nursing, Tokyo Medical and Dental University, Tokyo, Japan; 3 Department of Mental Health and Psychiatric Nursing, Osaka University, Osaka, Japan; Tribhuvan University Institute of Medicine, NEPAL

## Abstract

**Aim:**

Stigma against people who have alcohol and drug problems severely affects their health and well-being. An instrument based on stigma theory assessing individual-level stigma is essential for a comprehensive understanding of their stigma. We evaluated the validity and reliability of the Japanese version of the Substance Use Stigma Mechanism Scale (SU-SMS-J) among a population who had alcohol or drug use problems.

**Methods:**

Adults with experience in substance use disorders from psychiatry outpatient departments and rehabilitation facilities participated in the self-administered questionnaire survey. Confirmatory factor analysis was conducted to test the structural validity of the 5-factor model proposed in other language versions, and factor loadings and correlation between the subscales were confirmed. The correlations between the SU-SMS-J and psychometric properties related to substance use (e.g., severity of substance use, motivation to change) were investigated to assess concurrent validity. Internal consistency was assessed using Cronbach’s alpha coefficients.

**Results:**

Data from 126 participants were analyzed. The 5-factor model was acceptable with good or reasonable model fit indices. The correlations between subscales were weak to moderate, and this result suggested the SU-SMS-J assessed different but related components of stigma: enacted, anticipated, and internalized stigma from different stigma sources (family and healthcare workers). The SU-SMS-J and subscales showed moderate concurrent validity. Internal consistency was mostly sufficient, with Cronbach’s alpha coefficients of 0.86 for all items and 0.66–0.93 for subscales.

**Conclusions:**

The SU-SMS-J is valid and reliable for use among populations with substance use problems in various settings in Japan.

## Introduction

Substance use disorders consistently rank among the most stigmatized characteristics among various stigmatized physical and mental conditions worldwide [1, 2]. Stigma toward substance use is fostered by multi-level processes, including intimate relationships, decisions by social and health agencies, and governmental policy decisions [1]. Stigma toward substance use leads to poor health and well-being among people with substance use problems. Such persons may hesitate to seek treatment and support because they fear judgment from the people around them [3]. Stigma also affects their families and friends. Families and friends tend to feel limited in their ability to get help for their loved ones and feel shame, guilt, anxiety, or blame [4]. Family engagement and support is a key facilitator of substance use disorder (SUD) prevention, treatment, and recovery; however, people with SUD experienced social rejection from family members, including not answering phone calls, not being allowed into their homes, and even being disowned [5, 6]. Consequently, social rejection may lead to self-isolation, which causes re-engagement in substance use [5]. Previous reviews revealed that health professionals generally had a negative attitude toward patients with SUD [7, 8]. Thus, stigma from healthcare workers may also act as a barrier to seeking treatment for SUD. The structural and social negative labeling of people who use alcohol and other drugs can devalue people with substance use problems and make a social consensus that this population is not worthy of protection or opportunities to address their condition and should be penalized through institutionalized systems, policies, and practices [9]. Additionally, social-level stigma can restrict harm reduction practices that encourage justice and human rights in this population [9]. Social level stigma enormously impacts the lives of people with substance use problems because it affects life chances, such as employment opportunities, housing, and social interaction, and it perpetuates social, economic, and health inequities in this population [10]. Eventually, people with alcohol and drug problems feel cut off and isolated, lose their sense of self-worth or self-esteem, and feel marginalized.

The Stigma Framework, conceptualized based on existing stigma theory, is recommended for assessing individual-level stigma [9]. The Stigma Framework distinguishes among measurable stigma mechanisms from the stigma theory, including enacted stigma, anticipated stigma, and internalized stigma [11]. Enacted stigma is defined as personal experiences of stereotypes, prejudice, and discrimination from others in the past or present [12]. Anticipated stigma is defined as the extent to which a person with substance use problems expects to be the target of stereotypes, prejudice, or discrimination in the future [12]. People with substance use problems may anticipate stigma as a result of their own past experiences and also as a result of observing the experiences of others and/or being aware of societal stigma towards the population. Both enacted stigma and anticipated stigma reflect interpersonal processes experienced in relations with other people. Among people with substance use problems, families and healthcare workers are considered as significant others who may convey stigma toward them and influence their health and well-being [9]. Internalized stigma represents the endorsement and the application of negative briefs and feelings about people with substance use problems to the self [9, 12]. Internalized stigma sometimes refers to self-stigma, which reflects the application of negative stereotypes and prejudice to oneself [12].

Studies evaluating stigma among people with substance use problems have increased. However, a previous systematic review pointed out that most studies have not described the definition of stigma [13], and theory-based measurement evaluating comprehensive individual-level stigma, including enacted, anticipated, and internalized stigma, was limited [9]. To deal with this research gap, the Substance Use Stigma Mechanism Scale (SU-SMS) based on the Stigma Framework was developed by Smith et al. [9]. The original English version of the SU-SMS was developed corresponding to the HIV Stigma Mechanism Scale and included enacted, anticipated, and internalized components [9]. The enacted and anticipated assessed stigma from family and healthcare workers, which were common stigma sources most likely to influence people with substance use problems [5, 14]. Turkish and Spanish versions have been developed and adopted to people with substance use problems in different cultural backgrounds [15, 16]. English, Turkish, and Spanish versions were validated in the United States, Turkey, and Mexico, respectively, among people with various substance use problems, such as alcohol, marijuana, cocaine, opioids, and methamphetamine [9, 15, 16]. There are several scales assessing self-stigma among people with severe mental illness in Japan [17, 18]. However, these scales did not adapt to substance use disorder, and there has not been a Japanese version of a scale assessing individual-level stigma adapted to people with substance use problems based on the Stigma Framework. This study aimed to evaluate the validity and reliability of the Japanese version of SU-SMS among people experienced alcohol and drug use disorder.

## Materials and methods

### Participants and procedure

We recruited people with experience in substance use disorders aged 20 and over from psychiatry outpatient departments and facilities providing rehabilitation services for drug use problems in the Kanto region, Japan. We excluded people judged as therapeutically unsuitable for this study by their psychiatrist or facility staff due to health issues, such as severe withdrawal symptoms and physical or mental comorbidities. The minimum sample size was 100 for factor analysis based on a guide for developing health-related measurements [19]. We recruited about 130 participants, considering missing values.

A self-administered questionnaire was conducted among the participants who agreed to the study and provided informed consent. The participants chose paper-pencil or online surveys based on their preferences. After the participants answered the paper-based questionnaire, the researcher directly collected the questionnaires to maintain confidentiality. Data was collected from January 1st to December 31st, 2020.

### Development of the Japanese version of the SU-SMS

The SU-SMS consists of 18 items with a 5-point Likert scale, ranging from 1 (never/very unlikely/strongly disagree) to 5 (very often/very likely/strongly agree). A higher score indicates the individual feels more significant substance use stigma. A 5-factor model was suggested to capture the three stigma mechanisms (2nd order factors: enacted, anticipated, internalized) from the two external stigma sources (1st order factors: family and healthcare workers) [9]. The 5-factor model demonstrated good structural and concurrent validity and internal consistency among patients with alcohol and other drug use problems in a methadone maintenance clinic or HIV care clinic [9].

We referred to the guidelines for scale translation and cultural adaptation by Wild et al. [20] when translating the SU-SMS and evaluating its validity and reliability. The original version of the SU-SMS was translated into Japanese from English by two researchers independently after obtaining permission to translate and use the SU-SMS from the original author. Mental health professionals who trained for the treatment of substance use disorder (nurses and psychiatrists, including one native speaker of Japanese and English) checked the literal and conceptual equivalence between the original SU-SMS and the Japanese version and revised minor expressions of the words. The researchers reached a consensus that the Japanese version reflected the literal and conceptual content of the original SU-SMS. We finalized the Japanese version of the SU-SMS (SU-SMS-J, S1 Table).

### Measures for testing validity

#### Severity of substance use disorder

Alcohol Use Disorders Identification Test (AUDIT) [21, 22] and Drug Abuse Screening Test (DAST) [23, 24] were used to assess the severity of alcohol and drug use disorder, respectively. The validity and reliability of the Japanese versions of AUDIT and DAST were confirmed in previous studies [22, 24]. The total score summed up each item response (after revising the invert scale in DAST), and higher scores in both assessment instruments indicate more severe conditions of alcohol and drug use problems. We hypothesized that the SU-SMS-J was positively correlated with AUDIT or DAST.

#### Motivation to change

Previous studies revealed that self-stigma leads to passivity in behavioral change [25]. Motivation to change was measured with the Stage of Change Readiness and Treatment Eagerness Scale-8 version for Alcohol/Drug Use (SOCRATES-8A/8D) [26, 27]. The validity and reliability of the Japanese version of SOCRATES-8A/8D were confirmed in a previous study [27]. The total score summed up each item’s response after revising the invert scale, and higher scores indicate a higher motivation to change. SOCRATES-8A/8D has three subscales: recognition, ambivalence, and taking steps. We hypothesized that SU-SMS-J was negatively correlated with higher motivation change: the total and three subscales’ scores of the SOCRATES-8A/8D.

#### Duration of treatment and self-help group

We evaluated the correlation between the SU-SMS-J score, psychiatric treatment duration (total years and months), and duration of self-help group participation (total years and months). We hypothesized that SU-SMS-J was positively correlated with a long duration of treatment and self-help group participation because people with serious self-stigma are likely to drop out from the treatment and self-group activity. There were some missing values for the questions about psychiatric treatment or self-help group because participants who had not received psychiatric treatment or had not participated in the self-help group did not answer these questions.

*Types of substance and psychiatric comorbidity*. Drug use problems are generally considered more stigmatized than alcohol use problems [1], especially in countries where the drug policy is based on zero tolerance, as is the case in Japan. The type of primary substance and comorbidity were self-reported (alcohol or drug). Additionally, people with comorbidity for substance use disorder and other psychiatric disorders are likely to be more stigmatized [28]. We hypothesized that people with drug use problems or comorbidity had higher scores for SU-SMS-J than people with alcohol use problems or without comorbidity. We asked whether the participants had received treatment for psychiatric disorders other than substance use disorder or not at the time of the survey.

#### Demographic variables

We collected demographic variables and information on substance use history: sex (male, female, other), age, educational attainment (junior high school, high school, vocational college, or bachelor’s degree or higher), employment (currently employed or unemployed), marital status (currently married or single), age of first primary substance use, age of substance use problem cognition (when the participant recognized his/her substance use problems), age of first psychiatric treatment (if applicable), days of substance use in the past month, comorbidity of psychiatric disorder (received treatment for psychiatric disorders or not at the time of the survey), use of medical treatment (yes or no), duration of medical treatment (if applicable, total years and months), participation in a self-help group (yes or no), and duration of self-help group participation (if applicable, total years and months).

### Statistical analysis

We excluded participants with missing values for SU-SMS-J or primary substance. First, we summarized the participants’ demographic variables by type of primary substance. Second, to test the structural validity of SU-SMS-J based on the 5-factor model proposed in the original English, Spanish, and Turkish translated versions, confirmatory factor analysis was conducted using structural equation modeling. We considered correlated error variance between enacted and anticipated stigma from the same stigma source (i.e., enacted and anticipated stigma from family, enacted and anticipated stigma from healthcare workers), which was reported in the original version. Model fit was assessed using the Root Mean Square Error of Proximation (RMSEA; < 0.07), Comparative Fit Index (CFI; > 0.90), and Tucker-Lewis Index (TLI; > 0.90). Means, standard deviations of each item, and standardized factor loadings for the 5-factor model were calculated. We confirmed the factor correlations between subscales using Pearson’s correlation coefficient. Third, to evaluate concurrent validity, we assessed the correlation between SU-SMS-J and AUDIT/DAST-10, SOCRATES-8A/8D, and duration of treatment and self-help group using Pearson’s correlation coefficient. Additionally, we compared the scores of SU-SMS-J by types of substance and psychiatric comorbidity using t-test. Finally, we calculated Cronbach’s alpha coefficient to evaluate internal consistency. Cronbach’s alpha coefficients more than or equal to 0.70 were considered satisfactory [29]. All statistical analyses were performed using Stata 16 for Windows (StataCorp L.P., College Station, TX).

### Ethical consideration

We explained the aims, study procedure, the voluntary nature of participation, and anonymity. Written informed consent was obtained in a document or online format. The study protocol was approved by the ethics committee of Tokyo Medical and Dental University (No. M-2019-104).

## Results

### Participant characteristics

We excluded six responses because of missing data for SU-SMS-J or primary substance, and the remaining 126 participants (alcohol: n = 68, drug: n = 58) were included in the analyses. Table 1 shows participants’ characteristics and background of substance use problems. As for recruitment settings, 80 (63.5%) were from psychiatric outpatient departments, and 46 (36.5%) were from rehabilitation facilities. About 80% of the participants were male, and the mean age was 46.4 (SD = 12.1). Average days of substance use in the past month were 3.8 days (SD: 8.0). The mean age of first primary substance use was 19.5 (alcohol: 15.9, drug: 23.6). About 40% of the participants had psychiatric comorbidity. Participants who had used psychiatric treatment comprised 105 (83.3%). About 60% participated in self-help groups.

**Table 1:** Participant characteristics by primary substance (N = 126).

		Total	%/SD	Alcohol	(n = 68)	Drug	(n = 58)
		n/mean		n/mean	%/SD	n/mean	%/SD
Recruitment setting	Psychiatric outpatient department	80	63.5%	57	83.8%	23	39.7%
	Rehabilitation facility	46	36.5%	9	13.2%	35	60.3%
Sex	Male	105	83.3%	56	82.4%	49	84.5%
	Female	15	11.9%	10	14.7%	5	8.6%
	Other	6	4.8%	2	2.9%	4	6.9%
Age		46.4	12.1	50.8	12.4	41.2	9.4
Educational attainment	Junior high school	16	12.7%	3	4.4%	13	22.4%
	High School	47	37.3%	27	39.7%	20	34.5%
	Vocational college	13	10.3%	6	8.8%	7	12.1%
	Bachelor’s degree or higher	50	39.7%	32	47.1%	18	31.0%
Employment	Currently employed	47	37.3%	27	39.7%	20	34.5%
Marital status	Currently married	36	28.6%	27	39.7%	9	15.5%
Age of first primary substance use		19.5	8.0	15.9	3.9	23.6	9.4
Age of problem cognition		32.4	11.8	35.2	13.1	29.2	9.2
Age of first medical treatment		38.6	13.6	43.0	14.7	33.3	10.0
Days of substance use in the past month		3.8	8.0	4.7	8.7	2.7	6.9
Comorbidity of psychiatric disorder		52	41.3%	27	39.7%	25	43.1%
AUDIT (n = 55)				17.2	11.0		
DAST-10 (n = 58)						4.8	3.1
Use of psychiatric treatment		105	83.3%	56	82.4%	49	84.5%
Duration of psychiatric treatment (years, n = 52)		4.8	5.1	5.6	7.0	4.1	2.7
Participation in self-help group		77	61.1%	34	50.0%	43	74.1%
Duration of self-help group participation (years, n = 31)		4.7	4.8	6.7	6.4	3.9	3.9

AUDIT: Alcohol Use Disorders Identification Test. DAST: Drug Abuse Screening Test.

### Structural validity

**Fig 1:**
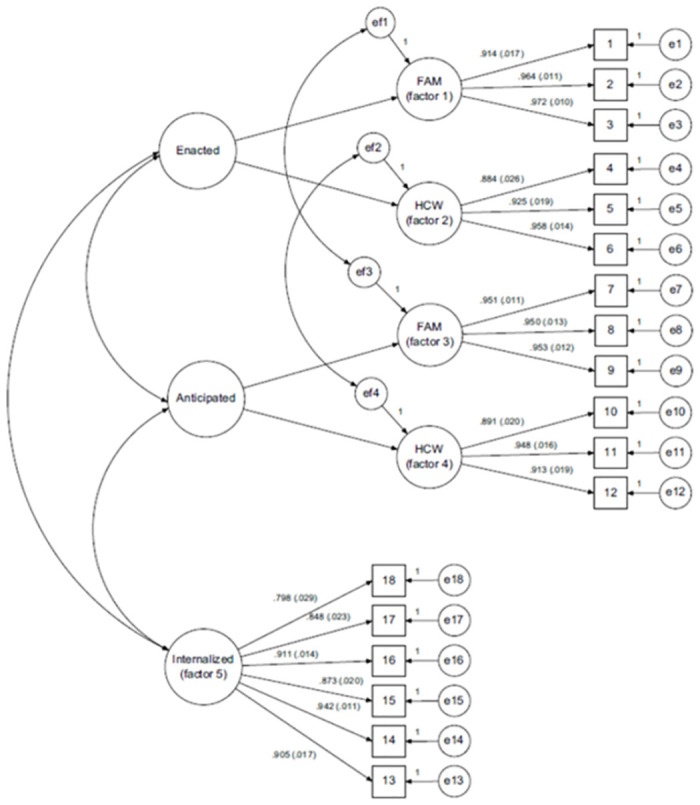
Structural validity of the 5-factor model based on the original SU-SMS using structural equation modeling. FAM: Family members’ stigma source, HCW: Health care workers’ stigma source.

The results of the structural validity of SU-SMS-J based on the 5-factor model are shown in Fig 1 and Table 2. The model fit indices were RMSEA = 0.090 (90% confidence interval: 0.074–0.106), CFI = 0.911, and TLI = 0.892. Similar to the original SU-SMS, the model fit was improved when considering correlated error variance between enacted and anticipated stigma from the same source. The means, standard deviations of each item, and standardized factor loadings are presented in Table 2. Except for one item (#5), the factor loadings were over 0.40. All factor loadings were significant at < 0.001. The floor effect was observed at items included in the subscales of enacted and anticipated stigma from healthcare workers (#4, 5, 6, 10, 11, and 12). Scores on the internalized stigma subscale were higher than scores on enacted or anticipated stigma subscales. Moreover, subscale scores from family were higher than from healthcare workers for enacted and anticipated stigma subscales.

**Table 2:** Structural validity and internal consistency of the five-factor factor model of the Substance Use Stigma Mechanism Scale (N = 126).

Factor			Item		Mean	SD	Factor loading	SE
	2nd-order	1st-order						
#1	Enacted (α = 0.75)	FAM (α = 0.86)	1	Family members have thought that I cannot be trusted.	4.2	1.2	0.75	0.05
			2	Family members have looked down on me.	3.5	1.5	0.91	0.03
			3	Family members have treated me differently.	3.2	1.5	0.83	0.04
#2		HCW (α = 0.66)	4^a^	Healthcare workers have not listened to my concerns.	2.1	1.2	0.83	0.06
			5^a^	Healthcare workers have thought that I’m pill shopping, or trying to con them into giving me prescription medications to get high or sell.	1.5	1.1	0.35	0.09
			6^a^	Healthcare workers have given me poor care.	1.8	1.0	0.79	0.06
#3	Anticipated (α = 0.84)	FAM (α = 0.93)	7	Family members will think that I cannot be trusted.	3.3	1.3	0.91	0.02
			8	Family members will look down on me.	2.9	1.4	0.92	0.02
			9	Family members will treat me differently.	3.0	1.4	0.89	0.02
#4		HCW (α = 0.80)	10^a^	Healthcare workers will not listen to my concerns.	1.9	1.0	0.72	0.06
			11^a^	Healthcare workers will think that I’m pill shopping, or trying to con them into giving me prescription medications to get high or sell.	1.7	1.0	0.73	0.06
			12^a^	Healthcare workers will give me poor care.	1.7	1.0	0.82	0.05
#5	Internalized (α = 0.91)	SELF	13	Having used alcohol and/or drugs makes me feel like I’m a bad person.	3.0	1.3	0.66	0.06
			14	I feel I’m not as good as others because I used alcohol and/or drugs.	3.1	1.3	0.65	0.06
			15	I feel ashamed of having used alcohol and/or drugs.	3.0	1.3	0.76	0.04
			16	I think less of myself because I used alcohol and/or drugs.	2.8	1.3	0.91	0.02
			17	Having used alcohol and/or drugs makes me feel unclean.	2.6	1.3	0.87	0.03
			18	Having used alcohol and/or drugs is disgusting to me.	2.9	1.4	0.86	0.03

FAM: Family members’ stigma source, HCW: Health care workers’ stigma source

All factor loadings were significant at < 0.001.

α: Cronbach’s α. Cronbach’s α for all items was 0.86.

^a^: Floor effect (mean–SD < 1) was observed.

Table 3 shows the correlations between subscales. Enacted and anticipated stigma had significantly medium positive correlations (r = 0.39–0.64). Internalized stigma did not correlate significantly with enacted stigma, while it had weak correlations with anticipated stigma (r = 0.20–0.25). For enacted and anticipated subscales, the correlations of subscales between stigma from family and healthcare workers were relatively weak (r = 0.19–0.23).

**Table 3:** Correlation of factors between subscales of the Japanese version of the Substance Use Stigma Mechanism Scale (N = 126).

Factor		Enacted			Anticipated			Internalized
		Total	FAM	HCW	Total	FAM	HCW	Total
Enacted	Total	1.00						
	FAM subscale	0.87***	1.00					
	HCW subscale	0.71***	0.27**	1.00				
Anticipated	Total	0.61***	0.56***	0.39***	1.00			
	FAM subscale	0.58***	0.64***	0.23*	0.90***	1.00		
	HCW subscale	0.39***	0.19*	0.49***	0.74***	0.38***	1.00	
Internalized	Total	0.07	0.04	0.09	0.25**	0.22*	0.20*	1.00

FAM: Family members’ stigma source, HCW: Health care workers’ stigma source

Correlation is significant at 0.001 (***), 0.01 (**), or 0.05 (*).

### Concurrent validity

The results of concurrent validity are presented in Tables 4 and 5. The anticipated stigma score was significantly correlated with the AUDIT score (r = 0.27). The total and internalized scores were correlated with the DAST score (r = 0.28 and 0.32, respectively). As for motivation to change, the total, enacted, and anticipated stigma were negatively correlated with the taking steps subscale of SOCRATES-8A. However, contrary to our hypothesis, the internalized stigma score was positively correlated with the recognition subscale of SOCRATES-8A. There was no significant association between SU-SMS-J scores and the DAST score. The duration of psychiatric treatment was negatively correlated with the anticipated subscale score (r = -0.34). The duration of self-group participation was negatively correlated with the total and anticipated subscale scores (r = -0.36 and -0.52, respectively). The total and enacted subscale scores among the participants with drug problems were significantly higher than those of the participants with alcohol problems. There was no significant difference in the scores of SU-SMS-J between participants with and without psychiatric comorbidity.

**Table 4:** Concurrent validity: Correlation with severity of substance use disorder, motivation to change, and duration of treatment or self-help group participation.

	Total	Enacted	Anticipated	Internalized
AUDIT (n = 55)	0.20	-0.03	0.27*	0.19
DAST-10 (n = 58)	0.28*	0.04	0.21	0.32*
SOCRATES-8A (n = 68)	-0.04	-0.18	-0.20	0.23
Recognition	0.13	-0.10	-0.11	0.39**
Ambivalence	0.08	0.02	-0.01	0.13
Taking step	-0.27*	-0.34**	-0.33**	0.03
SOCRATES-8D (n = 58)	-0.01	0.07	-0.03	-0.06
Recognition	0.01	0.10	-0.03	-0.04
Ambivalence	0.24	0.20	0.19	0.14
Taking step	-0.21	-0.08	-0.18	-0.19
Duration of psychiatric treatment (n = 52)	-0.18	-0.07	-0.34*	-0.02
Duration of self-help group participation (n = 31)	-0.36*	0.18	-0.52**	-0.24

AUDIT: Alcohol Use Disorders Identification Test. DAST: Drug Abuse Screening Test.

SOCRATES: The Stages of Change Readiness and Treatment Eagerness Scale.

Correlation is significant at 0.01 (**), or 0.05 (*).

**Table 5:** Concurrent validity: Differences by types of substance and psychiatric comorbidity.

	Alcohol	Drug	p ^a^	Comorbidity (-)	(+)	p ^a^
	(n = 68)	(n = 58)		(n = 71)	(n = 52)	
	Mean (SD)	Mean (SD)		Mean (SD)	Mean (SD)	
Total	46.0 (1.5)	50.5 (1.6)	0.02	46.8 (1.3)	49.9 (1.9)	0.09
Enacted	15.4 (0.6)	17.1 (0.7)	0.03	15.7 (0.6)	16.9 (0.8)	0.09
Anticipated	13.9 (0.7)	15.4 (0.7)	0.06	13.9 (0.6)	15.4 (0.8)	0.06
Internalized	16.8 (0.8)	18.1 (0.9)	0.13	17.3 (0.7)	17.6 (1.0)	0.40

^a^: t-test

### Internal consistency

The results of internal consistency are shown in Table 2. Cronbach’s alpha coefficient for all items of the SU-SMS-J was 0.86. Cronbach’s alpha coefficients for the subscales were above 0.70 (α = 0.75–0.93), except for the subscale of enacted stigma from healthcare workers (α = 0.66).

## Discussion

This study investigated the validity and reliability of SU-SMS-J, which assesses individual-level stigma among a Japanese population with substance use problems. The findings suggested that SU-SMS-J had reasonable structural and concurrent validity and internal consistency. However, some results were contrary to our hypotheses, and some differences from the results of the original SU-SMS were demonstrated.

As for structural validity, the 5-factor model proposed by the other language versions was acceptable with good or reasonable model fit indices. However, the results of the model fit were not as good as the original SU-SMS [9]. Additionally, one item had an insufficient factor loading at 0.35 (item #5: Healthcare workers have thought that I’m pill shopping, or trying to con them into giving me prescription medications to get high or sell). Participants’ characteristics might cause this result. The number of participants who misused prescription drugs might be small because more than half of the participants had alcohol problems, not drug problems, and 60% of the participants with drug problems were recruited from rehabilitation facilities. Most people living in rehabilitation facilities were likely to be methamphetamine users rather than prescription drug users [30]. Therefore, most of the participants did not have problems related to prescription drugs and might not have recognized self-stigma related to prescription drug problems. Recently, however, prescription drug abuse has been a major problem recently around the world [31, 32], especially in female patients [32]. Because the percentage of female participants was small in this study, self-stigma related to prescription drug problems could be underestimated. Additionally, some people might have both alcohol and prescription drug problems at the same time or one after another [33]. It was difficult to decide whether item #5 is not appropriate for people with alcohol use disorder in this study. Further studies, including those with more female patients recruited from different settings, are necessary to confirm the appropriateness of item #5.

Moreover, the results showed that the items reflecting self-stigma from healthcare workers were low and had a floor effect. This was also possibly due to participants’ characteristics. The psychiatry outpatient department, as the recruitment setting in this study, had specialized treatment for substance use disorder. The healthcare workers in the recruitment settings were likely to be well-trained and had the necessary skills to create therapeutic alliances with the patients. Additionally, staff in rehabilitation facilities usually provide service users support to create positive relationships with healthcare workers. Accordingly, most participants were likely to be satisfied with their relationship with healthcare workers, and they might not feel self-stigma caused by them. Even though there were floor effects, we considered that these items were essential to assess comprehensive individual-level stigma and should not be removed because the tendency for the scores on the enacted and anticipated stigma from healthcare workers to be lower than those from family was also observed in the development study of the original SU-SMS [9].

The findings from the correlations between subscales suggested that each subscale was related to the other, but these subscales are distinct as three components of stigma. Internalized stigma was not correlated with enacted stigma, but only with anticipated stigma. Further qualitative and quantitative study is needed to investigate the relationship between enacted and internalized stigma. Weak correlations between stigma from family and healthcare workers were observed for enacted and anticipated subscales. Additionally, the mean scores of enacted and anticipated subscales from family members were higher than those from healthcare workers. Because a similar tendency was reported in the original SU-SMS [9, 16], this finding suggested that people with substance use problems might had different experiences of enacted and anticipated stigma from family and healthcare workers. Countries with a cultural background of institutional collectivism, like Japan, are considered more likely to have stigma toward SUD [34]. Thus, people with SUD and their families in this study might be affected by social-level stigma, and the participants could have more negative relationships with their families [35]. Families that are highly influenced by social-level stigma could criticize family members who use substances and exacerbate their social isolation and self-stigma [35–37]. Although there were some limitations to the results of structural validity, the 5-factor model based on the original SU-SMS was thought to be adaptable to the Japanese population with substance use problems.

Concurrent validity was reasonable because our hypotheses were supported to some extent. The severity of substance use problems was positively related to high individual-level substance use stigma assessed by SU-SMS-J. High motivation to change for alcohol problems, long duration of psychiatric treatment, and self-help group participation were negatively associated with high individual-level substance use stigma. These findings were consistent with the original SU-SMS [9]. Individual-level stigma among people with drug use problems was more severe than in people with alcohol problems. Moreover, individual-level stigma among people with drug use problems was more severe than for people with alcohol problems. However, motivation to change for drug problems was not related to substance use stigma. This might be because of the small sample size of the participants who had drug use problems and psychiatric comorbidity. There was no correlation between individual-level stigma and psychiatric comorbidity, but this result was consistent with the previous study [9].

High to moderate internal consistency of the SU-SMS-J was achieved across all items (Cronbach’s alpha = 0.86) and subscales (Cronbach’s alpha = 0.66–0.93). Only the internal consistency for the subscale of enacted stigma from healthcare workers was insufficient. This result might be influenced by item #5, which had low factor loading and floor effect. The Cronbach’s alpha coefficients were about the same as the results of English and Spanish versions [9, 16].

There were some limitations to this study. First, the generalizability of the findings might be limited because of the small sample size and selection bias. The participation of females and people with prescription drug problems were limited. Further evaluations of the validity and reliability of the SU-SMS-J will be needed with a larger sample of substance users with various characteristics, such as females and patients with prescription drug problems. Second, we did not confirm test-retest reliability. Third, the questionnaire was self-reported. Some variables, such as psychiatric comorbidity, might not be correctly answered.

## Conclusions

SU-SMS-J was developed to assess three distinct stigma mechanisms (enacted, anticipated, and internalized stigma) from different stigma sources (family and healthcare workers) among a Japanese population with substance use problems. The moderate validity and reliability of the SU-SMS-J were confirmed in this study. SU-SMS-J helps comprehensively understand individual-level stigma among Japanese people with substance use histories and can evaluate the effects of interventions to reduce stigma toward substance use.

## Supporting information

S1 Table The Japanese version of the Substance Use Stigma Mechanism Scale (SU-SMS-J). (PDF)
