# Supplementary material for: Validity and reliability of the Japanese version of the Substance Use Stigma Mechanism Scale
Source: PLoS One. 2024 Oct 31;19(10):e0310514. doi: 10.1371/journal.pone.0310514 (PMC11527305; doi:10.1371/journal.pone.0310514)
Supplement: S1 Table — (PDF) [file pone.0310514.s001.pdf]

以下に続く質問では、あなたのアルコールもしくは薬物またはその両方の使用歴について、過去または現在の使用経験も含めてうかがいます。それぞれの質問について考え、あなたの答えに〇をつけて下さい。最初の質問では、あなたのアルコールまたは薬物、もしくはその両方の使用歴に対して、**過去に**人からどのように扱われたかについてうかがいます。２番目の質問では、あなたのアルコールまたは薬物、もしくはその両方の使用歴に対して、**これから先**人からどのように扱われると思うかについてうかがいます。

あなたのアルコールまたは薬物、もしくはその両方の使用歴に対して、**過去に**、どのくらいの頻度で以下のような扱いを受けましたか？答えに〇をつけてください。

		一度もない	そんなにない	少し	しばしば	何度も
1)	家族に自分は信用できないと思われた	1	2	3	4	5
2)	家族から見くだされた	1	2	3	4	5
3)	家族から自分だけ違う扱いを受けた	1	2	3	4	5
4)	医療従事者は私の心配事を聞いてくれなかった	1	2	3	4	5
5)	医療従事者に、ハイになったり、転売したりするために、私が処方薬を買いあさっている、もしくは嘘をついて処方せんをもらおうとしていると思われた	1	2	3	4	5
6)	医療従事者からひどい対応を受けた	1	2	3	4	5

あなたのアルコールまたは薬物、もしくはその両方の使用歴に対して、**これから先**、以下のような扱いを受ける可能性はどれくらいありそうですか？

		まったくなさそう	ありそうもない	どちらとも言えない	ありそう	とてもありそう
7)	家族に自分は信用できないと思われるだろう	1	2	3	4	5
8)	家族は自分のことを見下すだろう	1	2	3	4	5
9)	家族から自分だけ違う扱いをされるだろう	1	2	3	4	5
10)	医療従事者は私の心配事を聞いてくれないだろう	1	2	3	4	5

		まったくなさそう	ありそうもない	どちらとも言えない	ありそう	とてもありそう
11)	医療従事者に、ハイになったり、転売したりするために、私が処方薬を買いあさっている、もしくは嘘をついて処方せんをもらおうとしていると思われるだろう	1	2	3	4	5
12)	医療従事者からひどい対応を受けるだろう	1	2	3	4	5

あなたは自分のアルコールまたは薬物、もしくはその両方の使用歴ついて、どのように感じていますか？

		全くそう思わない	そう思わない	どちらとも言えない	そう思う	とてもそう思う
13)	アルコール・薬物の使用歴があることで、自分は悪い人間のように感じる	1	2	3	4	5
14)	アルコール・薬物を使っていたので、自分は他の人のように良い人ではないと思う	1	2	3	4	5
15)	アルコール・薬物を使用していたことを恥じている	1	2	3	4	5
16)	アルコール・薬物を使用していたので、ダメな人間だと思う	1	2	3	4	5
17)	アルコール・薬物を使用していたことで、自分がけがれているように感じる	1	2	3	4	5
18)	アルコール・薬物を使用していたことで、自分に嫌悪感を抱いている	1	2	3	4	5
